# Surgical Hip Dislocation for Management of Acetabular Osteochondroma in an Adult

**DOI:** 10.1155/2017/8481563

**Published:** 2017-07-25

**Authors:** Alexandre H. Nehme, Georges F. Haidamous, Hicham G. Abdelnour, Jad N. BouMounsif, Joseph W. Wehbe, Ramzi C. Moucharafieh

**Affiliations:** Department of Orthopedic Surgery and Traumatology, Saint Georges University Medical Center, Balamand University, P.O. Box 166378, Achrafieh, Beirut 1100 2807, Lebanon

## Abstract

The purpose of this study is to report a rare case of acetabular osteochondroma with a unique clinical presentation occurring in an adult with normally developed hips. The distinctive size and location of the lesion required an open approach with surgical dislocation of the hip for complete resection.

## 1. Introduction

Osteochondromas are considered the most common benign bone tumors [1, 2]. They are cartilage capped bone exostoses affecting all regions of the skeleton and arise from the growth plates of long bones and usually develop in relation to the periosteum [1, 3]. They can be sessile or pediculated [1]. They emerge as solitary lesions due to somatic mutation or as multiple lesions in the context of systematic disorders such as multiple hereditary exostoses (MHE) [2, 4]. Malignancy transformation is usually to chondrosarcomas and occurs in less than 1% of cases [1]. Most of the reported cases in the literature are extra-articular lesions more commonly on tubular bones, scapula, and iliac wing [1, 2]. Few studies reported the occurrences of these lesions as intra-articular and the occurrence in the acetabulum is considered rare. Many of the reported acetabular cases were in the pediatric population with MHE resulting in hip dislocation or dysplasia [5]. Some of these intra-articular osteochondromas are amenable to open surgical resection with hip dislocation [4] or to arthroscopic resection whether on the femoral side [2] or on the acetabular side [5].

The purpose of this study is to report a rare case of acetabular osteochondroma with a unique clinical presentation occurring in an adult with normally developed hips. The distinctive size and location of the lesion required an open approach with surgical dislocation of the hip for complete resection.

## 2. Case Presentation

A 26-year-old, white, male patient presented to the clinic for evaluation of left groin pain and progressive left hip stiffness. Pain started 9 months prior to presentation. Pain was increasing in intensity, moderately strong on presentation and without radiation. Pain was exacerbated by weight bearing and ambulation. Physical examination revealed a painful and limited left hip range of motion (ROM) with a flexion of 80°, adduction of 0°, abduction of 15°, external rotation of 30°, and internal rotation of 10°.

**Figure 1:**
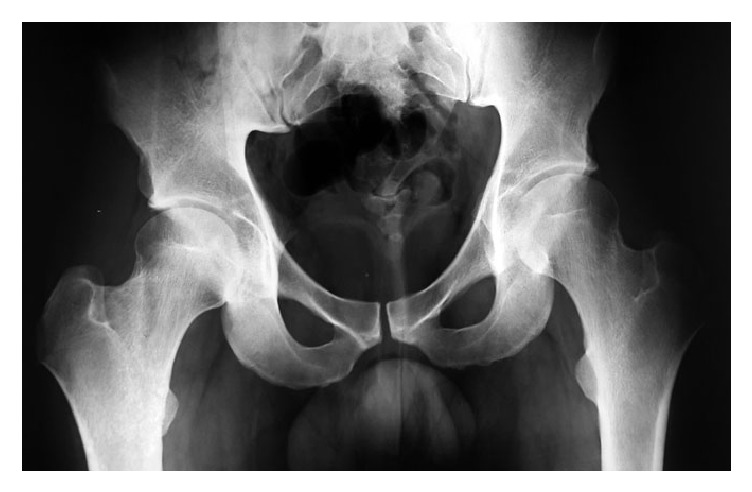
AP view radiograph of the pelvis revealing opacity in the left acetabular fossa.

Standard radiographs were initially performed including AP pelvis view that revealed the presence of an opacity in the left acetabular fossa (Figure 1).

**Figure 2:**
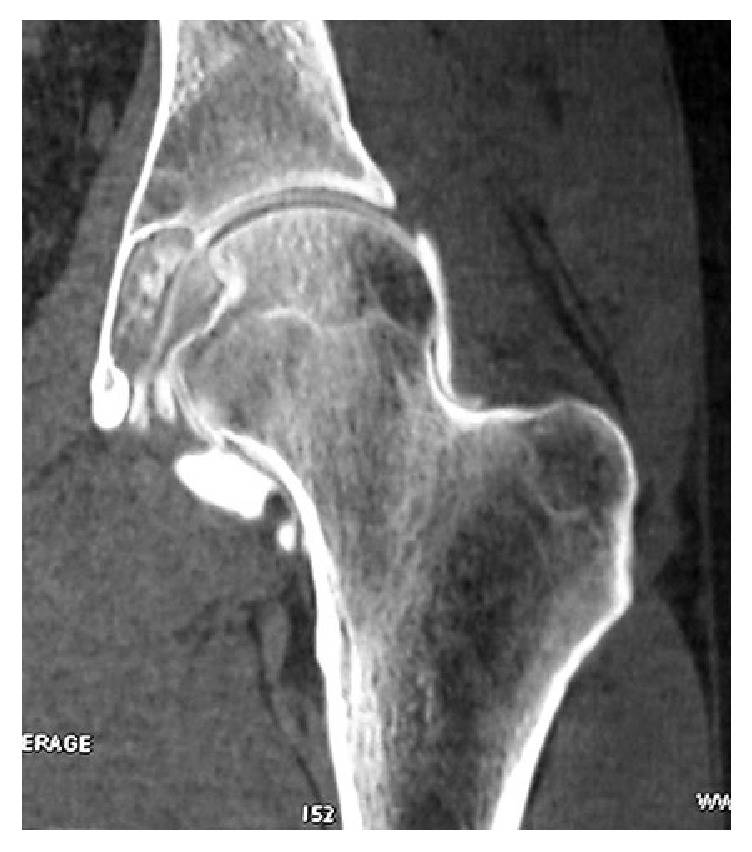
CT arthrogram revealing a lesion expanding medially through the acetabular fossa with sclerotic rim.

Therefore a CT arthrogram was ordered identifying a 4.5 × 2.4 × 1 cm lesion expanding medially through the acetabular fossa but respecting its inner table. The upper and lower limits of the lesion were, respectively, above and below the equator of the femoral head. The lesion was surrounded with a sclerotic bonny rim not deforming the inner acetabular wall thus representing a slowly growing lesion (Figure 2).

**Figure 3:**
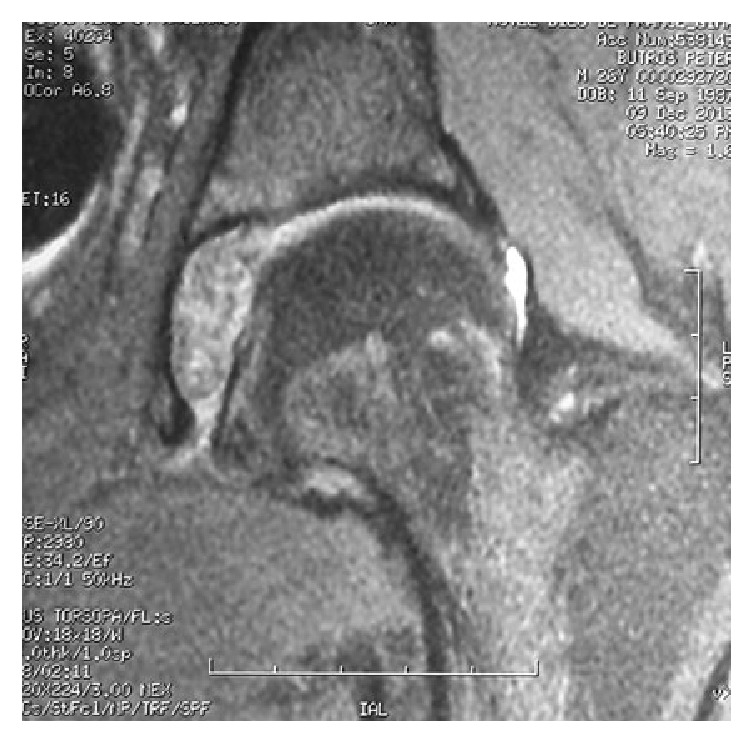
MR arthrogram showing a cavity in the acetabular fossa filled with a lesion with an isosignal similar to cartilage. The lesion is medially facing the fovea of the femoral head and indenting on the ligamentum teres.

For further evaluation, an MR arthrogram was done showing a cavity in the acetabular fossa filled with a lesion with an isosignal similar to cartilage highly suggestive of primary osteochondromatosis. The lesion is medially facing the fovea of the femoral head and indenting on the ligamentum teres (Figure 3).

A CT guided biopsy was not undertaken due to the benign nature of this lesion on different imaging modalities, associated with the fact of being already intra-articular. The patient was therefore hospitalized for an excisional biopsy. We felt at that time that the size of the lesion, its depth, and the fact that more than half of its volume was located below the femoral head equator make it not amenable to arthroscopic excision. An open approach with surgical hip dislocation was therefore carried out.

## 3. Surgical Procedure

**Figure 4:**
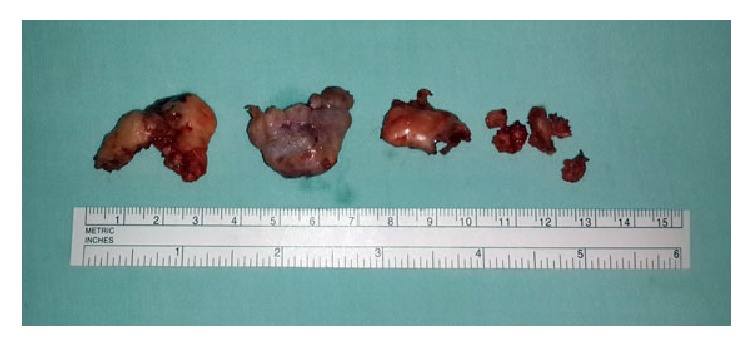
The excised three big osteochondral fragments with several smaller fragments.

A right hip arthrotomy was performed using a transtrochanteric anterior approach to the left hip and a Z-shaped capsulotomy as described by Ganz et al. [6]. Before performing the trochanteric osteotomy two holes were drilled preparing a place for the screws that will later reattach the greater trochanter. The hip was then dislocated anteriorly and the acetabulum was visualized entirely. No synovial effusion was found; the articular cartilage and acetabular labrum were intact. The lesion was accessed through the acetabular fossa. Three big osteochondral fragments were easily excised along with several smaller fragments (Figure 4).

**Figure 5:**
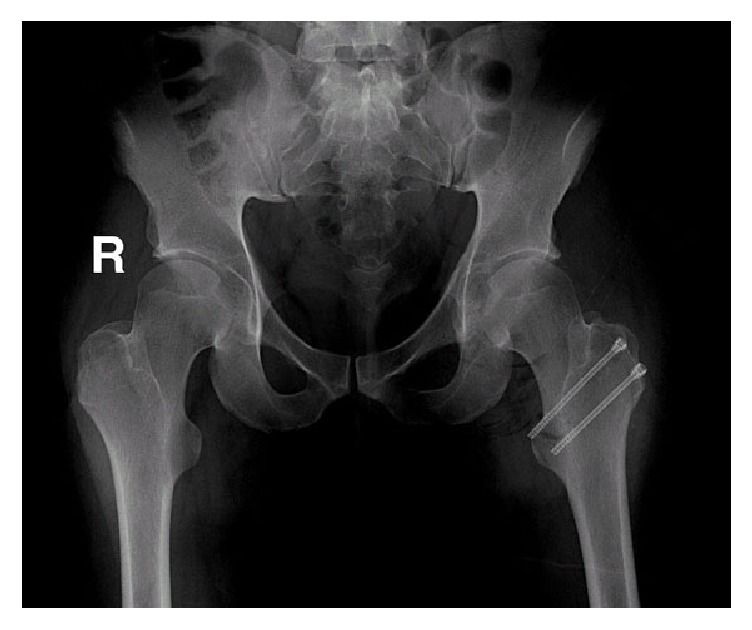
Postoperative radiographs showing the greater trochanter reattached using 2 cannulated 6.5 mm screws.

The sclerotic rim of the lesion was intact with no breaches in the medial wall. Burring was done to the sclerotic margins in order to allow adequate bone healing. Extensive lavage was then performed to remove all intra-articular debris. After relocating the hip and closing the capsule, the greater trochanter was reattached using 2 cannulated 6.5 mm screws (Figure 5).

All the excised fragments were sent to pathological analysis which confirmed the diagnosis of intra-articular acetabular osteochondroma.

The postoperative course was uneventful. The patient was allowed to ambulate on day two post-op with no weight bearing and two arm supports for 6 weeks. The patient was then discharged home on the third post-op day. Physical therapy exercises were started on week 2 post-op with full passive ROM exercises and isometric strengthening of the quadriceps. The patient was relieved from his preoperative pain with gradual improvement in his ROM. Follow-up radiographs at 6 weeks revealed complete healing of the trochanteric osteotomy and progressive return to full-weight bearing was authorized with progressive strengthening of the gluteus medius. On the 6 months' follow-up visit, the patient was found to be pain-free with normal ROM. 18 months later, the patient was still pain-free with normal left hip function.

## 4. Discussion

Extra-articular osteochondroma is the most common type of benign tumors of the bone. Solitary intra-articular acetabular osteochondromas is a very rare entity and all published cases were described in a pediatric population [4, 5, 7]. This is the first report to our knowledge describing an intra-articular acetabular osteochondroma in an adult with normally developed hips.

Previously reported cases in the pediatric population were either treated via open surgical hip dislocation with resection of the lesion or resected arthroscopically [4, 5, 7]. In our present case, arthroscopic resection was considered first but the location and the depth of the lesion and its protrusion through the acetabular fossa made us change our strategy.

In fact, when simulating arthroscopic traction of around 1.5 to 2 centimeters necessary to access the central compartment of the hip, an imaginary line drawn tangentially to the top of the femoral head, and representing the arthroscope, would still be in a position too proximal to get to the lesion for appropriate resection.

Thus we chose instead the transtrochanteric anterior hip dislocation technique as described by Ganz et al. which allowed complete access to the acetabulum [6]. This technique allowed safe intra-articular surgery bypassing the limitations and difficulties inherent to hip arthroscopy [6]. Iatrogenic injury to the cartilaginous surfaces of the femoral head and acetabulum is thus minimized without compromising the vascularity of the femoral head [6].
